# Editorial: Professional and vocational identity development

**DOI:** 10.3389/fpsyg.2024.1425138

**Published:** 2024-06-11

**Authors:** Eveline Wuttke, Karin Heinrichs, Stefanie A. Hillen, Kristina Kögler

**Affiliations:** ^1^Economics and Business Education, Goethe University Frankfurt, Frankfurt, Germany; ^2^Institute of Vocational Education, University of Teacher Education Upper Austria, Linz, Austria; ^3^Faculty of Humanities and Education, Department of Education, University of Agder, Kristiansand, Norway; ^4^Department of Vocational, Business and Technical Education, Institute for Educational Science, University of Stuttgart, Stuttgart, Germany

**Keywords:** professional identification, identity development, career development, professional development, vocational education

In recent research on career development, job retainment, and vocational education and training (VET), professional and vocational identification processes and identity development have proven to be key to forming a strong commitment to a profession and/or employer and to lead to better performance and satisfaction at work, resulting in fewer resignations (van Dick et al., 2004; van Dick, 2017). Empirical findings indicate that professional and vocational identification processes and identity development can be supported in experienced workers, novice workers, and students in vocational training programs. However, little is known about the conditions necessary for either explicitly promoting professional identification and identity development or how to provide adequate space for their development at different career stages (e.g., apprenticeship, professional development in mid-career, re-education, or career transitions). Therefore, this Research Topic deals with the development, predictors, and effects of vocational and organizational identification in VET in multiple domains and professions. The objective of this editorial is to provide an overview of the research perspectives and aims of the articles presented in this Research Topic. It will shed light on current theoretical approaches, empirical research on professional identity, and identification development in different countries, in a variety of professions, and across career stages.

Identification with the training company is a central goal of VET, not least because it should have positive effects on both the trainees and the training company. In their article, Maué et al. analyze the development, predictors, and effects of organizational identification among trainees in Germany. Organizational identification is defined as the extent to which trainees identify with the norms and values of their training company and feel part of the company. In their longitudinal study, the authors focus on the first year of training. Their findings indicate that organizational identification has a positive influence on emotional commitment and trainees' own assessment of their competencies. Strong organizational identification correlates with greater trainee retention.

According to Godfredson's Career Choice Theory, unconscious processes influence the development of young peoples' career aspirations. Mutlu et al. present a newly developed approval-sensitive career orientation workshop called “Logic of Career Choice”, which was designed to encourage youths to reflect on unconsciously developed career preferences and excluded career options. Results from their quasi-experimental study with a control and treatment group of more than 1,500 secondary students indicate that this workshop—moderated by decision-making certainty—can improve career choice by promoting reflection on needs and career-related activities. Thus, the study provides an impetus for designing approval-sensitive career orientation.

Teachers' identification with their profession is assumed to correlate with their professional behavior and desire to remain in the profession. In a longitudinal study (four measurement points, 79 vocational teachers in Germany) Weiß et al. investigated two conditions of teachers' identification development during teacher training: perceived autonomy support and autonomy thwarting behavior of seminar teachers. Cross-lagged panel analyses show that professional identification after 6 months of teacher training significantly predicts the intention to remain in the teaching profession half a year later. Significant cross paths describe positive effects between professional identification and autonomy support, and negative effects between professional identification and autonomy thwarting.

Vocational identification, crucial for both individuals and organizations, denotes alignment with one's career and employer. It fosters positive emotions and job satisfaction for employees and enhances performance and bolsters retention for employers. Amid demographic shifts and declining full-time work, securing young talent early and preventing attrition during apprenticeship is vital. Research underscores the correlation between vocational identification and satisfaction, retention, and performance. However, scant attention has been paid to apprentices' identification in vocational programs. In the study by Wuttke et al. factors affecting apprentices' identification are explored, particularly training quality and trainer competence. Results indicate that strong identification with both the career and the organization is largely driven by quality training and job satisfaction.

Thole's contribution outlines a mixed methods approach to identity development in VET in Germany. Through analysis of longitudinal case studies and curricular documents, the impact of a new VET curriculum for the retail sector on learners' vocational identity is explored and current research designs and relevant theoretical approaches are reviewed. Data collection and analysis culminate in a theme-centered process analysis. Exemplary cases demonstrate the effectiveness of the approach in understanding individual identities and generating broader insights into the identity development of VET learners. The potential of the approach for pedagogical design support is discussed.

Summarizing these research-based discussions, it is important to keep in mind that for companies, recruiting and retaining well-trained workers has become a major challenge that is expected to continue in the foreseeable future. Many economies are struggling with shortages in qualified labor force, which will intensify drastically in the coming years (Cedefop, 2016, p. 59). Identification therefore is a key objective of all VET programs (Heinrichs et al., 2022). In this time of instability, as industries face rapid technological advances and changing economic landscapes, identification with training institutions and occupations will be both challenging and of crucial importance.

## Author contributions

EW: Writing – original draft. KH: Writing – original draft. SH: Writing – original draft. KK: Writing – original draft.
